# Flexible and navigable suction access sheaths: what size stone particles can be cleared?

**DOI:** 10.1111/bju.16844

**Published:** 2025-06-29

**Authors:** Richard Menzies‐Wilson, Jessica Williams, Koushikk Ayyappan, Thijs Ruiken, Candace Rhodes, Ben Turney

**Affiliations:** ^1^ Nuffield Department of Surgery University of Oxford Oxford UK; ^2^ Boston Scientific Corporation Marlborough MA USA

**Keywords:** flexible and navigable suction access sheaths, stone‐free rate, SFR, clearance, ureteroscopy

## Abstract

**Objectives:**

To perform benchtop experiments on flexible and navigable suction (FANS) ureteric access sheaths (UASs) to determine the clearance capabilities for various stone sizes when paired with different‐sized ureteroscopes.

**Methods:**

Quartz stones were used as a surrogate for renal stones. Stone samples were created to simulate the range of particle sizes produced by ‘dusting’ a 1‐cm calcium oxalate monohydrate stone. The stone mixture was introduced into an open vial at a 30° angle in aliquots every 3 min over the course of 30 min, mimicking their gradual production by lasertripsy. This benchtop model focused on the effects of geometry (between FANS access sheath and ureteroscope) and suction, excluding complex fluid flow. An 11/13‐F ClearPetra® FANS UAS with an indwelling ureteroscope was free to move in the vial and targeted visible stones. The vial was intermittently filled to 40 mL with water and emptied using 200‐mmHg suction. The experiment had two arms: (1) a permanently indwelling ureteroscope in the FANS UAS to mimic continuous lasering with aspiration and (2) intermittent withdrawal of the ureteroscope to mimic pausing lasering to clear larger fragments through the empty FANS sheath lumen. Three ureteroscope sizes were used: 9.5‐F (Lithovue™), 7.5 F (PUSEN) and 6.3 F (Hugemed). The experiment was performed four times for each ureteroscope. [Correction added on 4 July 2025, after first online publication: In the preceding sentence, “three” has been corrected to “four” in this version.]

**Results:**

With a permanently indwelling 9.5‐F ureteroscope, the FANS UAS cleared 64% of the overall stone mass but became blocked after an average of 21 min. Intermittent withdrawal of the ureteroscope cleared all stones. With indwelling 7.5‐F and 6.3‐F ureteroscopes, the FANS UAS did not become blocked and completely cleared stones of up to 500 μm and 2 mm, respectively.

**Conclusions:**

Without withdrawing the ureteroscope (potentially allowing continuous lasertripsy), it is possible to continuously aspirate small fragments alongside a 7.5‐F and a 6.3‐F ureteroscope in an 11/13‐F FANS UAS. Intermittent withdrawal of a ureteroscope from the FANS UAS allows complete stone clearance.

**Abbreviations**
FANS: flexible and navigable suction
SFR: stone‐free rate
UAS: ureteric access sheath

## Introduction

Retrograde intrarenal surgery is the first‐line treatment for <2 cm upper urinary tract stones [1]. Traditional laser lithotripsy and basketing techniques often leave behind small residual stone fragments (<2 mm), which may be a nidus for regrowth and require further intervention [2, 3]. As a result, in recent years, there have been several advancements in suction technology capable of improving the removal of residual stone material, which may increase stone‐free rates (SFRs).

Flexible and navigable suction (FANS) ureteric access sheaths (UASs) are a novel type of access sheath. They differ from traditional UASs in two distinct ways. Firstly, the proximal tip is flexible and so can be navigated to the stones within the renal calyces. Secondly, they have an outflow suction port, allowing negative pressure to aspirate stone dust and fragments. As with traditional access sheaths, the ureteroscope is passed through the FANS UAS lumen.

While it is possible to withdraw the ureteroscope before suctioning, leaving an empty FANS UAS lumen, this is time‐consuming and depletes the irrigation fluid in the renal pelvis, requiring re‐distension and navigation back to the treatment site. A more efficient approach would be to aspirate stone particles through the FANS UAS with the ureteroscope in place, minimising ureteroscope withdrawal. This approach could allow for more continuous laser lithotripsy under direct visualisation, whilst simultaneously clearing the resulting stone particles.

There have been case studies [4, 5] detailing the efficacy of FANS in improving SFRs and intra‐operative complication rates, and recently, some prospective, multicentre trials [6, 7]. However, the field has only begun to characterise the functionality of FANS access sheaths and techniques for their use, and there is still potential to further increase utility and efficacy [8, 9].

**Fig. 1:**
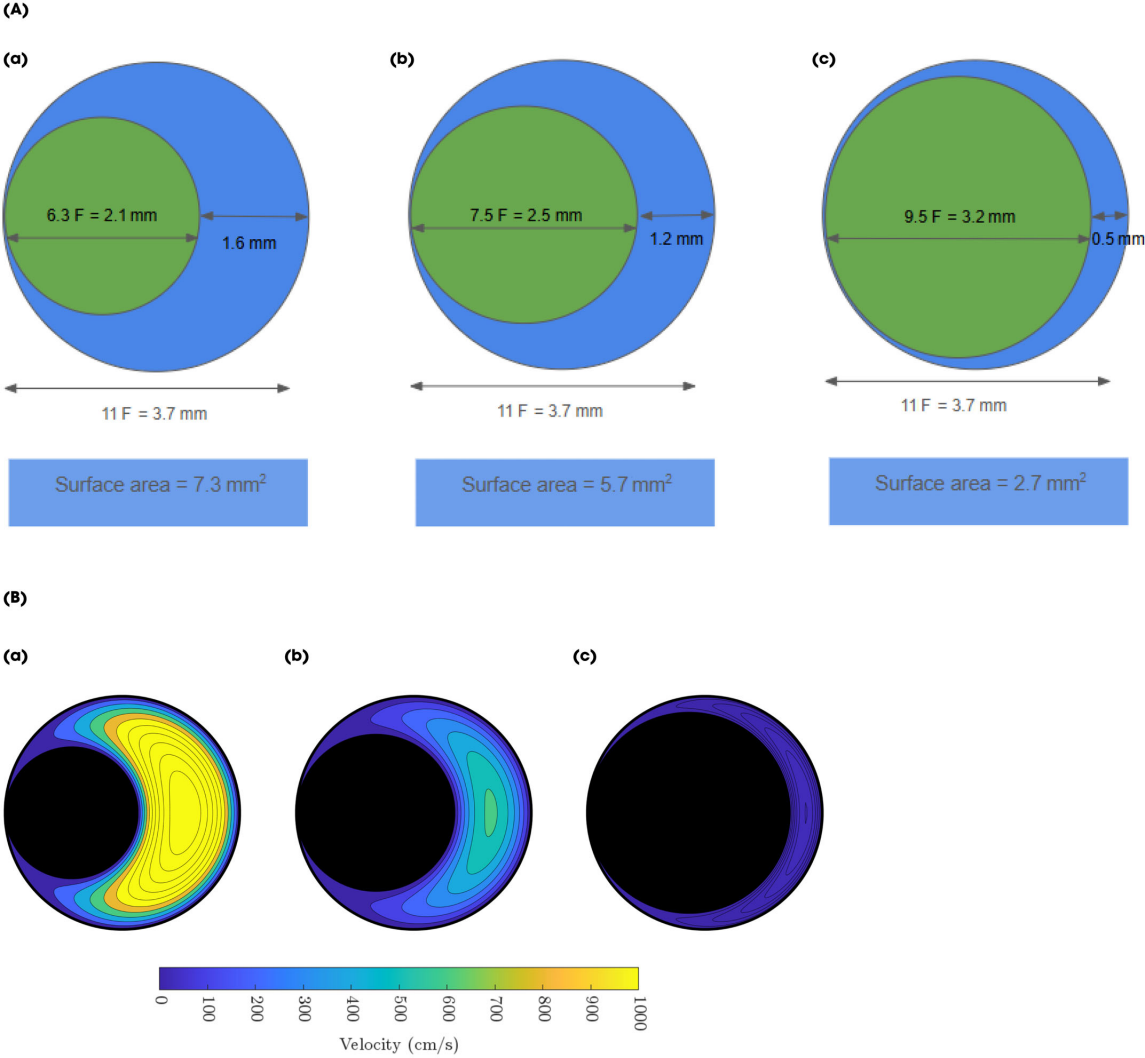
(**A**) Comparison of cross‐sectional areas of outflow tract through an 11/13‐F flexible and navigable suction (FANS) ureteric access sheath (UAS) with different‐sized indwelling ureteroscopes. The blue boxes show the surface area available for stone aspiration. (a) 6.3‐F ureteroscope (green circle) in a 11/13‐F FANS UAS (blue circle). (b) 7.5‐F ureteroscope in an 11/13‐F FANS UAS. (c) 9.5‐F ureteroscope in an 11/13‐F FANS UAS. (**B**) Comparison of cross‐sectional mathematically predicted fluid velocities through an 11/13‐F FANS UAS with different‐sized indwelling ureteroscopes. The velocities are with 200‐mmHg suction pressure. (a) 6.3‐F ureteroscope in an 11/13‐F FANS UAS; (b) 7.5‐F ureteroscope in an 11/13‐F FANS UAS; (c) 9.5‐F ureteroscope in an 11/13‐F FANS UAS.

We conducted benchtop experiments to evaluate the feasibility of stone fragment aspiration with FANS alongside an indwelling ureteroscope and to examine how ureteroscope size influences the clearance of stone particles of varying sizes. Using a smaller ureteroscope increases the outflow cross‐sectional area available for FANS aspiration (Fig. 1), which we hypothesised would enhance particle clearance. To test this, we developed a simplified benchtop model that intentionally excluded the full fluid dynamic complexities of ureteroscopy, allowing us to isolate outflow cross‐sectional area as the sole variable under investigation.

## Methods

### Quartz Stone Model

A quartz stone model was used as a surrogate for renal stones, since quartz has a similar shape, insolubility and density (2.6 g/cm^3^ vs 1.7–2 g/cm^3^) to calcium oxalate monohydrate [10]. Quartz stones were sieved into groups by particle diameter: <63, 63–125, 125–250, 250–500, 500–1000 μm, and 1–2 mm. Stone samples were created with a mixture of quartz particles in a distribution of sizes, mimicking the particle sizes produced from lasering a 1‐cm calcium oxalate monohydrate stone with the Lumenis Pulse 120H Holmium Laser System [10]. The distribution of particles (with masses of each) was: <63 μm (0.55 g), 63–125 μm (0.15 g), 125–250 μm (0.05 g), 250–500 μm (0.05 g), 500–1000 μm (0.1 g), and 1000–2000 μm (0.1 g).

### FANS Set‐up

**Fig. 2:**
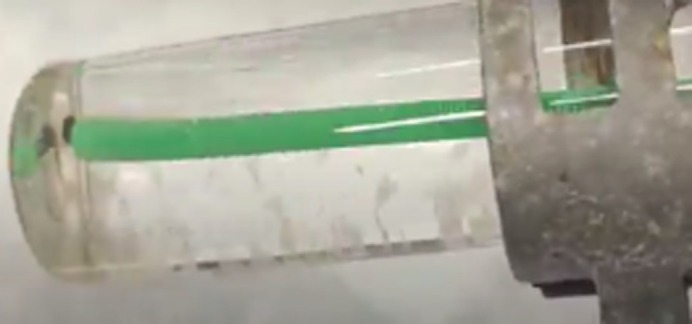
Experimental set‐up. Aspiration of stone fragments through an 11/13‐F ClearPetra flexible and navigable suction ureteric access sheath from a glass vial at a 30° incline.

A 11/13‐F 40‐cm ClearPetra® FANS UAS (Wellead, China) was placed within an open glass vial at a 30° angle, as shown in Fig. 1. A ureteroscope was placed within the FANS UAS, with its proximal end 2 mm beyond the tip of the UAS. Three ureteroscopes of different sizes were tested: 6.3‐F HugeMed (HugeMed, China), 7.5‐F PU3033A (Pusen, China) or 9.5‐F LithoVue™ single‐use digital flexible ureteroscopes (Boston Scientific, USA; Fig. 2). A 200‐μm MOSES™ single‐use holmium laser fibre was placed within the working channel.

Throughout the experiment, the irrigation was pressurised with an irrigation pressure bag to 200 mmHg and irrigation provided through the ureteroscope working channel. Suction of 200 mmHg was simultaneously continuously applied with a medical vacuum pump (SAM12; MGE Medical Supplies, UK) to the FANS UAS via a suction tube. By default, the FANS UAS vent was uncovered (open). Leaving the vent uncovered acted as an air off‐valve, with our previous work demonstrating that no suction is applied through the FANS UAS lumen; instead, ambient air is preferentially aspirated through the open vent [9]. The vial was filled to 40 mL water (to replicate a hydronephrotic kidney). Once 40 mL was reached the FANS UAS vent was covered and the (200‐mmHg) suction was applied to aspirate the fluid and particles through the vial through the FANS UAS. Once the vial was empty, the vent was uncovered and no further suction was applied, allowing the constant irrigation pressure to again refill the vial. This filling–emptying cycle was repeated continuously (not timed) from 0 to 40 mL throughout the experiment to replicate the periodic filling and draining of the renal collecting system during a case with the intermittent use of FANS.

To mimic the gradual production of particles during laser lithotripsy, the 1‐g stone fragment mixture was introduced into the vial in 10 aliquots, every 3 min, over a 30‐min period. The FANS UAS and indwelling ureteroscope were free to move in the vial to target visible stone particles. If the FANS UAS became blocked, the experiment was terminated. No mechanical manoeuvres were performed to try unblocking the FANS UAS.

As well as testing the three indwelling ureteroscopes, a positive control was performed with the 9.5‐F ureteroscope. The ureteroscope was intermittently withdrawn beyond the suction port once every 5 min whilst closing the vent and so applying suction, to allow the FANS UAS to empty. This mimics the pausing of lasering to aspirate larger residual fragments through an empty FANS UAS lumen.

After each experiment, the remaining stones in the vial were sieved, filtered and weighed to calculate the SFR (%) for each stone size category: %SFR = 1 − (residual mass/starting mass).

### Statistics

graphpad prism was used for statistical analysis. The total starting stone mass clearance was analysed using one‐way anova, and Tukey's post hoc was then used for increased statistical power (as *n* < 5 for each experiment). For clearance of particle sizes, two‐way anova was used, followed by Tukey's *post hoc* test. Statistical significance was set at *P* < 0.05 and all testing was two‐sided.

## Results

**Fig. 3:**
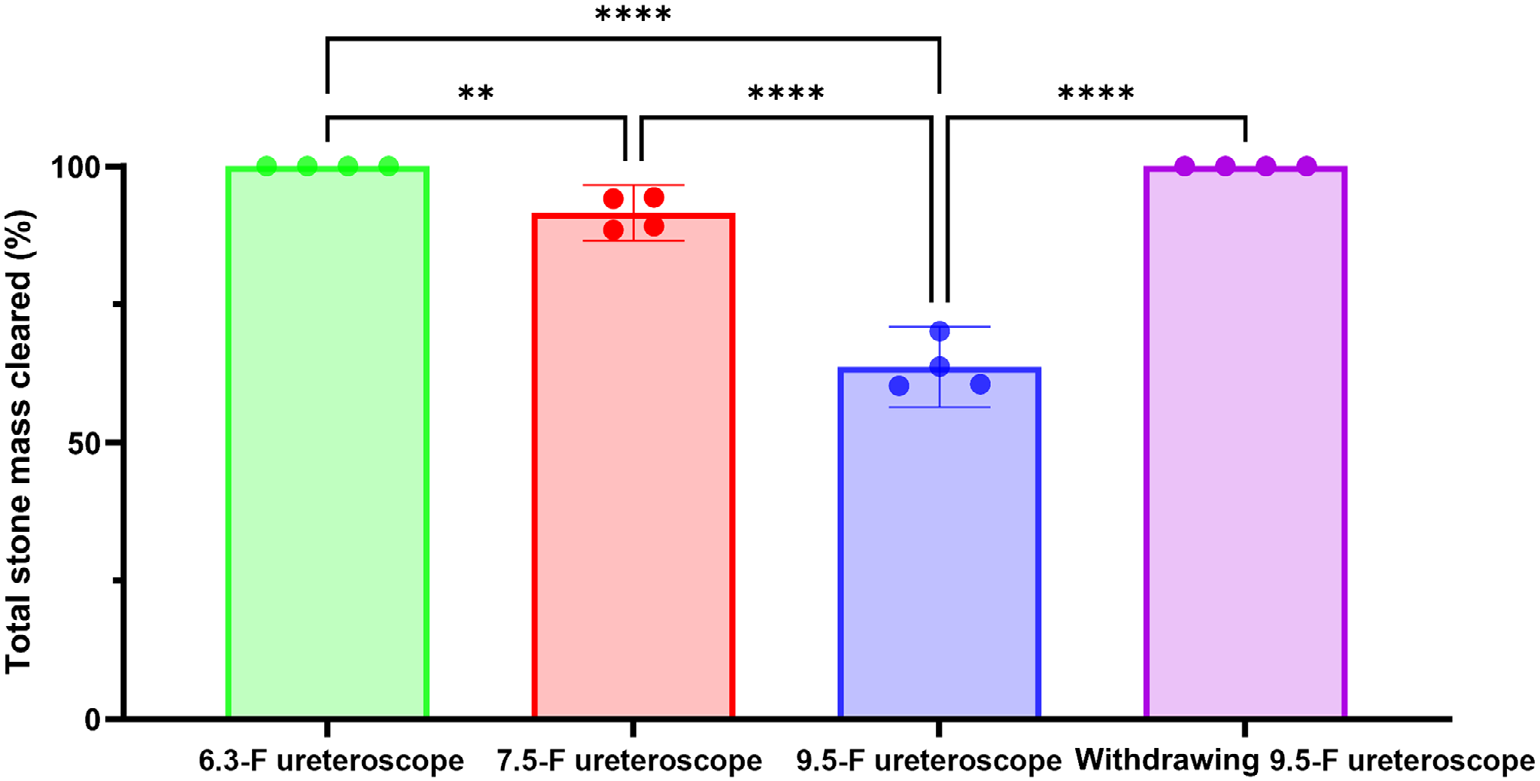
Total stone mass cleared (%) when using different sized ureteroscopes to suction through an 11‐F inner diameter ClearPetra flexible and navigable suction ureteric access sheath (*n* = 4) *P < 0.05, ****P < 0.0001. Data show mean ± 95% CIs.

With a permanently indwelling 6.3‐F ureteroscope, the FANS UAS cleared 100% of the overall stone mix across all particle diameters (Fig. 3). However, this fell to 92% with a 7.5‐F ureteroscope (*P* < 0.05), and 64% with a 9.5‐F ureteroscope (*P* < 0.001). Intermittently withdrawing the 9.5‐F ureteroscope every 5 min resulted in clearing 100% of the stone mix. The FANS UAS became blocked with the 9.5‐F ureteroscope after an average of 21 min (Fig. S1). The FANS UAS did not become blocked when using the other smaller ureteroscopes that were tested.

**Fig. 4:**
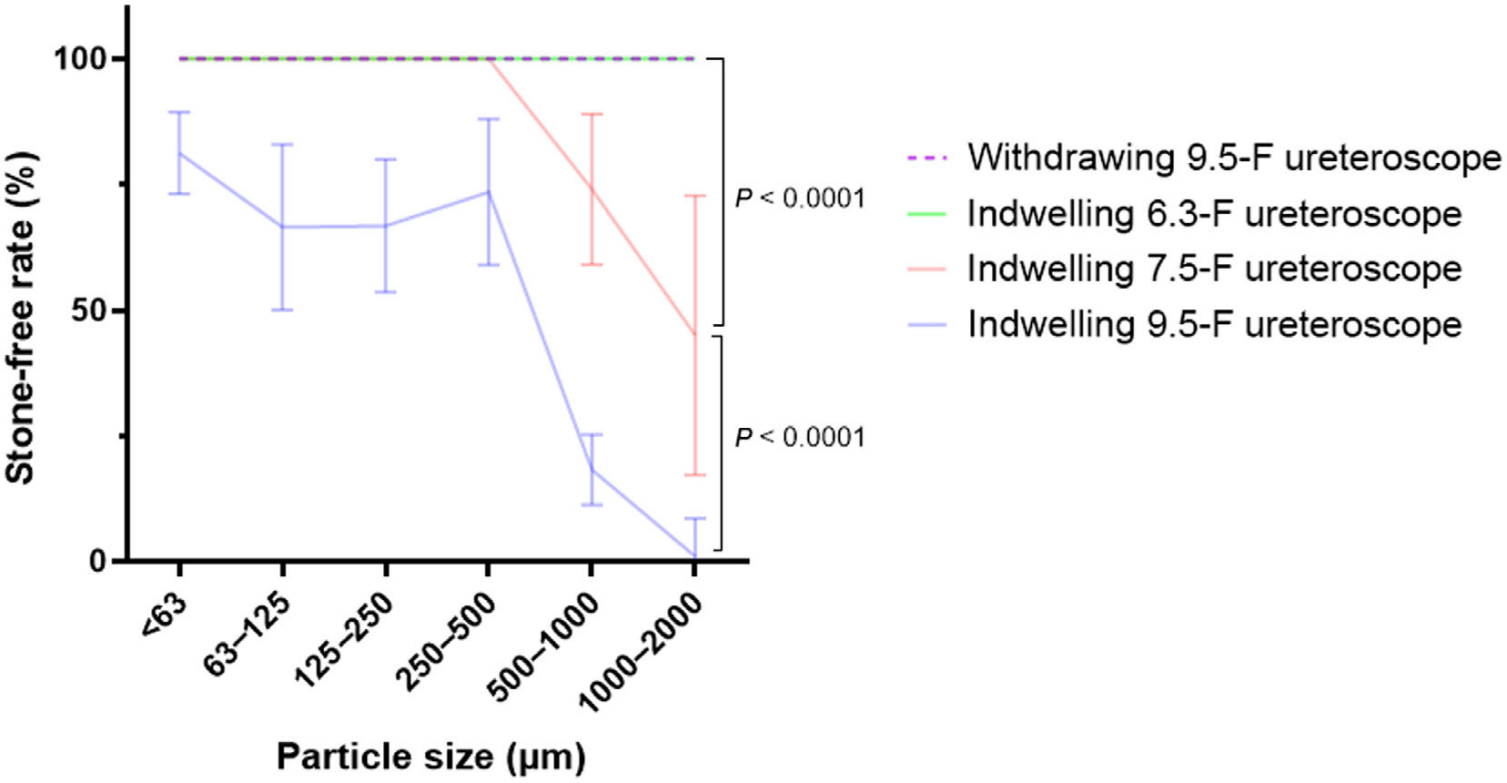
Flexible and navigable suction (FANS) during laser lithotripsy: stone‐free rate (%) through an 11/13F FANS ureteric access sheath alongside indwelling ureteroscopes of different sizes and ureteroscope withdrawal. (*n* = 4) Data shown as mean ± 95% CIs.

When suctioning with the ureteroscope within the FANS UAS, we observed a negative correlation between SFR and particle size (Fig. 4). Smaller ureteroscopes allowed aspiration of larger quantities of stone fragments of all sizes. For example, with the 9.5‐F ureteroscope, the SFR was 81% for particles <63 μm but only 1% for particles 1000–2000 μm. Similarly, with the 7.5‐F ureteroscope, the SFR was 100% for particles ≤500 μm, but only 45% for 1000–2000‐μm particles. With the 6.3‐F ureteroscope, the SFR was 100% for all particle sizes.

There was a significant difference in the SFR of particles 1000–2000 μm when comparing the indwelling 6.3‐F, 7.5‐F and 9.5‐F ureteroscopes: 100% vs 45% vs 1% (*P* < 0.0001). Intermittent withdrawal of the 9.5‐F ureteroscope, the positive control, cleared all stones and resulted in a 100% SFR.

## Discussion

FANS access sheaths are a novel innovation in ureteroscopy. Still relatively new to the market, there is a lack of consensus on the optimal techniques for their use, especially on when and how often during a procedure to utilise active aspiration. Many urologists currently withdraw the ureteroscope from the FANS sheath, leaving an empty lumen to aspirate out stone particles. Whilst this approach successfully clears stone particles through a wider outflow tract of only the sheath, it is time‐consuming and inefficient – laser lithotripsy needs to paused, the renal pelvis re‐distended and the stone position relocated to resume treatment. By contrast, particle aspiration while the ureteroscope is indwelling in the target treatment location with visualisation can facilitate more continuous laser lithotripsy.

Our results suggest that stone clearance alongside a permanently indwelling ureteroscope using a FANS UAS with active suction may be feasible. However, the diameter of particles cleared is influenced by the geometry of the outflow tract, specifically the cross‐sectional area between the outer diameter of the ureteroscope and the inner diameter of the FANS UAS (Fig. 1).

While the geometry of the outflow tract sets the outflow resistance for irrigation outflow, the size of stone particles that can pass out is much smaller than the maximum distance between scope and sheath. For a particle of any size to be carried in a current, the fluid velocity (Vf) needs to exceed the critical velocity (Vc) to carry the particle, as per Stoke's law:

Vf>Vc

The critical velocity to carry a particle is dependent on the particle diameter (*d*), along with other fixed variables: gravity (*g*), the density of the particle (*Pp*), the density of the fluid (*Pf*) and the viscosity of the fluid (*μ*):

$$\mathrm{Vc}=gd^{2}\left(\mathrm{Pp}-\mathrm{Pf}\right)/18\;\mu$$

The fluid velocity, for any given irrigation/suction pressure, is determined by the radius of the outflow tract (rt), that is, the cross‐sectional area between the ureteroscope and the FANS UAS, along with the length of the tube (*L*) and fluid viscosity (*μ*) as per Poiseuille's Law:

$$\mathrm{Vf}=rt^{2}/8\;\mathrm{\mu L}$$

Thus, the diameter of ureteroscope in a FANS UAS affects the radius of the outflow tract, having an exponential effect on the fluid velocity, at any given suction pressure. With a 6.3‐F ureteroscope in an 11/13‐F FANS UAS (at 200‐mmHg suction) the maximum fluid velocity is 1000 cm/s, compared with 600 cm/s with a 7.5‐F ureteroscope and 35 cm/s with a 9.5‐F ureteroscope. These fluid velocities can impact the size of stone particles carried by suction through the FANS UAS lumen. Increasing outflow cross‐sectional area increases critical fluid velocity and aligns with the empirical data of these configurations, allowing larger‐sized particles to be carried.

With currently available technologies (6.3‐F ureteroscope and 11/13‐F FANS UAS) it is possible to aspirate stone particles up to 2 mm through the FANS UAS alongside an indwelling ureteroscope. Whilst we demonstrated equal clearance by intermittently withdrawing a 9.5‐F ureteroscope, clearance alongside a ureteroscope potentially obviates the need for repeated withdrawals of the scope that may improve operative efficiency.

Whilst using a 7.5‐F ureteroscope it is still possible to leave the ureteroscope permanently in the 11/13‐F FANS UAS throughout the operation and aspirate alongside it. This clears all particles up to 500 μm (the particles which can disproportionately impact intra‐operative vision the most), leaving only the larger particles (>500 μm) to be aspirated at the end by withdrawing to leave an empty FANS lumen. It is these larger particles which have the lowest chance of spontaneous passage [11, 12, 13].

These results still support the possibility of using a 9.5‐F ureteroscope with an 11/13‐F FANS without blockage for up to 21 min. However, this combination may limit clearance of all particle sizes unless the ureteroscope is intermittently withdrawn, which facilitated complete clearance of all particle sizes. Since particle clearance is dependent on the outflow tract diameter (between ureteroscope and FANS UAS), combining a 9.5‐F ureteroscope with a larger 12/14‐F FANS UAS could further increase stone clearance without ureteroscope withdrawal.

There are several clinical studies evaluating the impact of varying outflow resistance on SFRs and subjective surgical performance [14, 15]. Kwok et al. [14] reported that the use of larger (11/13‐F or 12/13‐F) FANS sheaths vs smaller sheaths (10/12‐F) resulted in improved visibility but no difference in 30‐day SFR. Notably, this multicentre study did not use a standardised FANS technique. Geavlete et al. [15] reported in a single‐centre study that a 6.3‐F or 7.5‐F ureteroscope within a 10/12‐F FANS UAS resulted in SFRs of 95% vs 92.5% on CT at 30 days postoperatively, respectively.

Our study has several limitations. Firstly, the aspiration was performed in an open model at atmospheric pressure. This does not represent the complex fluid dynamics within a closed renal collecting system but instead offers a simplified model where the effect of ureteroscope and FANS sheath geometry on stone clearance could be assessed without other confounding factors. However, it is important to note that results from an open vial should be regarded as best‐case stone clearance with the ureteroscope/FANS sheath combinations – in clinical practice, particles are likely to be less easy to locate and blood or tissue may impact aspiration. Secondly, we used quartz as a proxy for renal stones. Whilst quartz has a similar density to calcium oxalate, and so is a reasonable proxy, it is not identical. In reality, there is a wide range of kidney stone compositions and densities. Only 36.2% of stones are pure calcium oxalate [16], and a large proportion of stones are a mix of compounds, such as 28.8% mixed calcium oxalate and phosphate. Given that the critical velocity to carry particles in a flow depends on the particle density, varying stone composition affects stone clearance. Hence our findings may not precisely represent the clearance characteristics of all types of kidney stones.

Further work is required to optimise and automate the best irrigation pressures, intrarenal pressures, flow rates and stone clearance rates for ureteroscopy with scopes, working channels and access sheaths of different geometries.

In conclusion, FANS UASs are a recent innovation in ureteroscopy, and there is a lack of evidence on optimal technique. While it is possible to withdraw the ureteroscope before suctioning, leaving an empty FANS UAS lumen, this is time‐consuming. A more efficient approach would be to aspirate stone particles through the FANS UAS with the ureteroscope in place, minimising ureteroscope withdrawal. This approach would allow for continued laser lithotripsy under direct visualisation whilst simultaneously clearing the resulting stone particles.

Our results suggest that stone clearance alongside an indwelling ureteroscope is feasible. However, the diameter of particles cleared is influenced by the geometry of the outflow tract, specifically the cross‐sectional area between the outer diameter of the ureteroscope and the inner diameter of the FANS UAS. This has an exponential effect on the fluid velocity through the FANS UAS, which determines the particle size that can be carried in the current.

It is possible to aspirate small particles (<500 μm) alongside a 7.5‐F ureteroscope in an 11/13‐F FANS UAS but larger particles would require withdrawal of the ureteroscope. Intermittent withdrawal of a 9.5‐F ureteroscope from the FANS UAS allows complete stone clearance, highlighting the importance of withdrawal when using larger ureteroscopes. It is possible to aspirate particles up to 2 mm alongside a 6.3 ureteroscope in an 11/13F FANS UAS.

## Disclosure of Interests

Bench test results may not necessarily be indicative of clinical performance. The testing was performed by or on behalf of Boston Scientific Corporation (BSC). Data on file. This study was funded by a research grant from BSC. The following authors are employees of BSC: Jessica Williams and Candace Rhodes. The following authors are recipients of research grant funding from BSC: Ben Turney and Richard Menzies‐Wilson. The following author has nothing to disclose: Koushikk Ayyappan.

## Supporting information


**Fig. S1.** Blocking time when using a permanently indwelling 9.5‐F ureteroscope to suction stones through an 11‐F FANS. Whiskers represent 95% confidence intervals. N.B. the other ureteroscope sizes did not incur blockages during the experiments.
