# Supplementary material for: Flexible and navigable suction access sheaths: what size stone particles can be cleared?
Source: BJU Int. 2025 Jun 29;137(Suppl 3):S71–7. doi: 10.1111/bju.16844 (PMC12950927; doi:10.1111/bju.16844)
Supplement: Supplementary file 1 — Fig. S1. Blocking time when using a permanently indwelling 9.5‐F ureteroscope to suction stones through an 11‐F FANS. Whiskers represent 95% confidence intervals. N.B. the other ureteroscope sizes did not incur blockages during the experiments. [file BJU-137-S71-s001.docx]

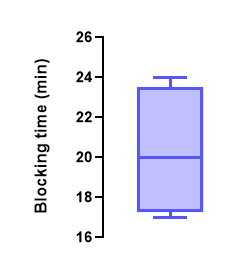


**Supplementary Figure 1: Blocking time when using a permanently indwelling 9.5F URS to suction stones through a 11F FANS.** Whiskers represent 95% confidence intervals.

N.B. the other ureteroscope sizes did not incur blockages during the experiments.
